# Extraction and injection of botulinum toxin with less wastage

**DOI:** 10.1111/srt.13816

**Published:** 2024-06-25

**Authors:** Kar Wai Alvin Lee, Lisa Kwin Wah Chan, Lee Cheuk Hung, Raymond Wu, Sky Wong, Jovian Wan, Kyu‐Ho Yi

**Affiliations:** ^1^ EverKeen Medical Centre Hong Kong Hong Kong; ^2^ Asia‐Pacific Aesthetic Academy Hong Kong Hong Kong; ^3^ Leciel Medical Centre Hong Kong Hong Kong; ^4^ Division in Anatomy and Developmental Biology Department of Oral Biology Human Identification Research Institute BK21 FOUR Project Yonsei University College of Dentistry Seoul South Korea; ^5^ Maylin Clinic (Apgujeong) Seoul South Korea

Dear Editor,

Botulinum toxin is one of the most popular non‐surgical cosmetic treatments worldwide.^1^, ^2^, ^3^, ^4^ It is used to relax facial muscles and reduce the appearance of wrinkles and fine lines. However, one of the major concerns associated with its usage is the wastage of the product.^5^ Botulinum toxin comes in a freeze‐dried form that has to be reconstituted with saline solution before being injected. Our usual practice often leads to significant wastage and increased expenses for practitioners and patients.

In recent years, there has been a growing interest in finding ways to reconstitute botulinum toxin with less wastage in order to have a cost‐effective treatment for patients. Several techniques have been proposed for this purpose, including removal of the vial cap^6^, rolling the vial between the palms of the hands for a minute prior to reconstitution^5^, and the use of specialized vials and syringes.^7^ In this article, we review the current state of knowledge about the extraction and administration of Botulinum toxin with less wastage and explore the potential benefits of these techniques for patients, and practitioners.

Keywords including “Botulinum toxin”, “Botox”, “Dysport”, “Xeomin”, “Siax”, “wastage”, “reduce wastage”, “lower the wastage” and “injection techniques” were used to conduct searches in the Ovid, PubMed, and MEDLINE databases. The aim was to identify relevant studies published in English between 2000 and 2023, focusing on botulinum toxin administration, wastage, and injection techniques. Subsequently, selected papers were further reviewed based on their relevance to our research topic. Finally, 11 papers were analyzed and classified according to the Oxford Center for evidence‐based medicine evidence hierarchy.

Niamtu et al.^8^ discuss the issue of neurotoxin waste during the extraction process of botulinum toxin from vials. The author notes that traditional methods of extraction may result in significant amounts of the product left in the vial stopper, therefore reducing the effective dosage received by the patient. The author suggests several techniques to minimize waste, including using a smaller gauge needle, drawing back on the plunger to flush out any trapped medication, and using a centrifuge to separate any trapped medication from the stopper. By adopting these methods, clinicians can maximize the amount of neurotoxin extracted from each vial and minimize waste. While the paper discusses an important practical issue, it is not based on empirical data or a systematic review of the literature and therefore represents the author's professional opinion rather than scientific evidence (Level 5).

Dykstra et al.^9^ tested various techniques for maximizing the extraction of botulinum toxin type A (BoNT‐A) from vials. They found that using a larger gauge needle and a more forceful aspiration technique resulted in significantly higher yields of medication. They also noted that using a mixing technique and taking care to avoid injecting air bubbles into the vial could further improve extraction efficiency. These findings have practical implications for clinicians who administer BoNT‐A injections. This study is a non‐randomized experimental study that evaluates the efficiency of different reconstitution methods for extracting BoNT‐A. While the study provides important information for clinicians using BoNT‐A, the evidence is not as strong as a well‐designed randomized controlled trial, which would be classified as Level 1 evidence. Additionally, the study is on an individual cohort, rather than a randomized sample of participants, which lowers the level of evidence further (Level IIb)

Solinsky et al.^6^ explore the problem of retained botulinum toxin after injections. While botulinum toxin has been shown to be effective in treating muscle spasticity and other disorders, it has also been reported that a significant amount of the toxin can remain in the needle or syringe after injection. This not only reduces the effective dosage received by the patient but also poses a potential safety risk, as the remaining toxin may accidentally be injected into the wrong site or person. The authors suggest several strategies for mitigating this problem, including using smaller gauge needles, flushing the syringe, and adjusting injection techniques. By adopting these measures, clinicians can improve both the safety and efficacy of botulinum toxin injections. This individual cohort study evaluated the efficiency of different techniques in reducing retained botulinum toxin post‐injection. While this study offers valuable insights for clinicians using botulinum toxin, the level of evidence is not as strong as a well‐designed randomized controlled trial (Level IIb).

Liu et al.^10^ research provides recommendations and current practices for the reconstitution and storage of BoNT‐A. The authors discuss key factors that can affect the efficacy and safety of BoNT‐A injections, such as pH, diluent type, and storage conditions. They also provide practical guidelines for preparing and storing BoNT‐A, including recommended dilution ratios, storage temperatures, and expiration dates. The paper is based on a literature review as well as input from a panel of experienced clinicians. Overall, the paper provides valuable guidance for clinicians who use BoNT‐A in their practice and aims to promote the safe and effective use of this medication (Level 5).

Kaplan et al.^11^ discuss the importance of accurate dosing and dilution of BoNT‐A when administering cosmetic injections. The author highlights the prevalence of improper dilution practices due to a lack of proper understanding of dosing calculations, which can result in adverse effects and suboptimal patient outcomes. The paper provides a step‐by‐step guide to accurately calculate and dilute botulinum toxins, emphasizing the need for thorough consideration of the patient's anatomy, desired outcome, and individual response to the treatment. Additionally, the authors highlight the importance of proper training, certification, and supervision for healthcare professionals administering botulinum toxin injections (Level 5).

Carruthers et al.^12^ present the consensus recommendations of a group of expert clinicians for the use of BoNT‐A in facial aesthetics. It covers a range of topics including the anatomy and physiology of facial muscles, the pharmacology and mode of action of BoNT‐A, patient selection and evaluation, dosing and injection techniques, and management of adverse events. The recommendations are based on a comprehensive review of the available literature and the collective expertise of the consensus group. The paper concludes with a summary of the key recommendations and guidelines for the safe and effective use of BoNT‐A in aesthetic applications (Level 5).

Foglietti et al.^7^study is a cross‐sectional study that primarily relied on a survey of the American Society of Aesthetic Plastic Surgery members. While it provides useful insights and perspectives on syringe types in botulinum toxin therapy from expert opinions, the study does not involve any interventions, direct comparisons, or establish causative relationships. Current evidence suggests that the cost per unit of botulinum toxin remains similar irrespective of the type of syringe used. However, the authors highlight the oversight of certain factors such as ergonomic design, dosage accuracy, and wastage in previous studies. Survey results indicate that while most surgeons do not favor a particular syringe type, those who do prefer the insulin syringe for its accuracy and cost‐effectiveness. Despite its limitations, this study offers valuable information for clinicians and researchers seeking a deeper understanding of the cost‐effectiveness of botulinum toxin therapy (Level 4).

Shetty et al.^13^ provide guidelines for the use of BoNT‐A in dermatological and cosmetic procedures. The authors highlight the importance of proper patient selection, dosage, and injection technique to achieve the desired outcomes while minimizing potential risks and side effects. The guideline covers several areas, including facial wrinkles, hyperhidrosis, chronic migraine, spasticity, and dystonia. The authors highlight the various injection techniques and precautions to be taken for each indication. The guideline provides useful recommendations for clinicians and specialists involved in the administration of BoNT‐A to improve their safety and efficacy profiles and to optimize patient outcomes (Level 5).

Mendez‐Eastman et al.^14^ offer a comprehensive review of BoNT‐A (BOTOX), covering both its medical and cosmetic applications. The review delves into its mechanism of action, pharmacology, dosage, and administration, with a strong emphasis on proper patient selection, informed consent, and injection technique to mitigate potential complications. Various medical indications such as spasticity, strabismus, and hyperhidrosis are described alongside their methods of administration, while potential adverse effects such as ptosis, diplopia, and flu‐like symptoms are also addressed. This review provides an insightful summary of BOTOX's efficacy and safety, aiding nurses, clinicians, and other healthcare professionals to understand the drug's clinical role and optimize treatment outcomes for patients. It is more of a narrative review without any formal systematic appraisal or synthesis of primary studies (Level 4).

Tarantino et al.^5^ introduce a novel technique for maximizing the use of botulinum toxin from 50‐unit vials. The standard protocol for the preparation of botulinum toxin solution involves reconstitution of the powder with saline and subsequent extraction of the desired dose. This results in a significant amount of waste as small remains in the vial after extraction. The authors suggest rolling the vial between the palms of the hands for a minute prior to reconstitution, which would result in a homogenous mixture and enable the complete extraction of the solution. This technique has demonstrated a 5%–12% increase in solution extraction compared to the standard protocol, leading to significant cost savings, particularly for clinics administering high volumes of botulinum toxin. Additionally, the technique eliminates the need for additional toxin vials, reducing the risk of cross‐contamination and upholding patient safety. In conclusion, rolling the vial between the palms prior to reconstitution may be an effective strategy to safely maximize botulinum toxin extraction, minimize wastage, and reduce costs (Level 5).

Bazargan et al^15^ conducted a prospective, single‐arm clinical trial to assess the effects of botulinum toxin injections on seborrheic dermatitis in 20 participants, using the Seborrheic Dermatitis Area and Severity Index for evaluation. Although there was a noted reduction in symptom severity in one‐month post‐injection, the improvement was not statistically significant. The authors advise against using botulinum toxin for seborrheic dermatitis based on these findings. They emphasize the importance of standardizing injection techniques and follow‐up protocols in future research. While previous studies suggested that botulinum toxin could reduce sebum production and alleviate symptoms, this study did not support those conclusions, potentially due to differences in follow‐up periods and injection methods. The authors suggest that intradermal injections might help reduce symptoms, whereas intramuscular injections could worsen them. They recommend further research with standardized methods and larger sample sizes to clarify the therapeutic potential of botulinum toxin for seborrheic dermatitis.

The injection of botulinum neurotoxin Type A is the most frequently performed aesthetic procedure worldwide.^16^, ^17^ While it is widely accepted that botulinum toxin diffusion occurs, the extent of its spread and its clinical significance remains contentious. Numerous factors are purported to influence this diffusion; however, identifying which factors hold the greatest clinical relevance is still a matter of considerable debate. In Brodsky et al.’s ^18^ study, the authors examine the extent and clinical significance of botulinum toxin diffusion and its influencing variables. Key factors include injection volume, concentration, dose, protein composition, and molecular size. The review of animal and human studies on different botulinum toxin products (onabotulinumtoxinA, abobotulinumtoxinA, incobotulinumtoxinA, and rimabotulinumtoxinB, letibotulinumtoxinA) shows that molecular weight and complexing proteins do not affect diffusion, whereas volume, concentration, and dose do.^19^, ^20^, ^21^, ^22^ The study notes that botulinum toxin products are not interchangeable, with some causing more diffusion‐related adverse events.

Techniques such as muscle biopsy and neural cell adhesion molecule staining confirm diffusion in both humans and animals, highlighting dose, concentration, and volume as major contributors. Other factors like needle gauge and injection method have less impact. Comparative trials indicate that abobotulinumtoxinA results in more adverse events in blepharospasm treatment, while rimabotulinumtoxinB shows greater diffusion and higher rates of dysphagia and dry mouth in cervical dystonia.

The paper emphasizes the need to understand different botulinum toxin serotypes, dosing variations, and side‐effect profiles to minimize adverse events. Accurate muscle targeting and precise dosing are suggested to control diffusion effects.

In clinical practice, employing a standard dilution with 2.5 mL of normal saline to use at a concentration of 4 U/0.1 mL, the botulinum toxin is observed to disperse approximately 1–3 cm in diameter from the point of injection. It is worth noting that while this observation is reported by the authors, it is not currently substantiated in the existing literature.

Based on currently available evidence, we outline the common methods of wastage of botulinum toxin during extraction and injection:

1. Wasting botulinum toxin by over‐injecting it in the desired locations.

Botulinum toxin is designed to address dynamic lines caused by movement but does not effectively treat static lines that persist over time. Many individuals may seek retreatment during their follow‐up sessions because they are disappointed with the outcome of their initial botulinum toxin treatment for wrinkles. Their disappointment arises from the persistence of static lines despite the inability of the treated muscles to contract.

2 Wasting botulinum toxin by injecting it in places where it is unnecessary.

Another common way to waste botulinum toxin is by treating lines that are caused by muscles elsewhere. For instance, the procerus muscle, responsible for causing a crease across the nose, might have been deactivated during the initial treatment. Nevertheless, it is common to observe instances of double treatment, where the muscle re‐treatedto address a different crease, often a deep vertical line, caused by the corrugator muscles pushing inwards. Another scenario involves the orbicularis oculi muscle, which is targeted during the treatment of crow's feet. Despite the initial treatment, clinicians may consider injecting additional botulinum toxin into the orbicularis oculi muscle during follow‐up appointments, even though the crease is caused by the zygomaticus major muscle.

Some patients may attempt to contract every facial muscle after receiving botulinum toxin injections in hopes of securing a top‐up during their follow‐up visit. While they are utilizing more muscles to form the wrinkles, physicians must be careful to prevent re‐treating muscles that have been previously treated muscles again, as it would result in wastage.

3 Wasting botulinum toxin by injecting it in ineffective locations.

Botulinum toxin can be wasted when injected into areas where it will not provide therapeutic benefits. A common example is the forehead aponeurosis, where injecting serves no purpose as there are no muscles present.

4 Dispensing excessive amounts of botulinum toxin into the air and discarding unused toxin remaining in the syringe.

Physicians or their team members may inadvertently contribute to botulinum toxin wastage during syringe preparation, especially if they are not responsible for purchasing or covering the costs. This could involve expelling product and air, resulting in wastage on the floor. To mitigate this, it is advisable to leave extra dead space in the syringe by pulling back the plunger before attempting to remove any trapped air bubbles. Careful maneuvering is required to collect the last bead of product from the needle before injecting it into the syringe.

Another common cause of botulinum toxin wastage is the dead space in the top of the syringe, where valuable product (approximately half a unit of botulinum toxin) may end up in the sharps container. This can be prevented by using syringes with minimal to no dead space.

5 Administering excessive botulinum toxin by inadvertently injecting it into the patient's face.

If physicians overlook the removal of tiny air bubbles from syringes during treatment preparation, valuable substances may be wasted. As the injection is administered, the compressed gas in these bubbles may expand due to the inertia of the rubber bung as the plunger is compressed. Consequently, upon withdrawing the needle after the injection, the decompressed needle may inadvertently expel botulinum toxin onto the patient's face, unintendedly targeting areas not originally intended for treatment. Despite the appearance of successful injection into the skin, up to 50% of the substance, particularly if the bubble is large, may resurface on the skin.

6 Leaving botulinum toxin in the vial or bottle to waste it.

To avoid wasting valuable botulinum toxin when discarding the container, the initial challenge is to ensure the transfer of every drop of product from the Botox vial into the syringes. Due to the complexity of this task, many individuals may overlook the need to leave any remaining substance behind.

### Recommendation

Physicians will almost always leave a sizeable amount within the vial if they prefer to withdraw directly through the vial stopper with a brand‐new needle. This was demonstrated in Niamtu et al.^8^ study that employed a random sample of “used and empty” vials and found that using this method to draw up the product might lose an average of five units of Botulinum toxin. By removing the rubber bung and syringing the remaining product directly, physicians can save some or all of the botulinum toxin (Level 2b). They should not use that needle to inject the patient since it will become very blunt and uncomfortable for your patients if it is forced against the glass of the vial. Since the needle is disposable, they could decide to bend it slightly to get closer to the liquid that is still to remove every drop (Level 5).

Use pliers or a specialized decapper to remove the vial's cap and bung in order to accomplish this (Level 2b). Then, tilt the vial toward that angle so you can see through the glass while looking down at the side of the label, and do so until you can see the little meniscus‐shaped collection of residual liquid at the glass's edge. Aspirate the liquid by inserting your needle into the glass with the bevel facing the glass (Level 5).

Another approach to reducing wastage involves the use of air displacement to remove excess liquid from the reconstituted Botox solution (Level 5). This technique involves drawing the solution into a syringe, compressing the plunger to remove excess liquid, and then injecting the product. The use of syringes with pre‐measured air chambers can help standardize the process and reduce the risk of over‐displacing the liquid.

Specialized vials and syringes have also been developed to help reduce Botulinum toxin wastage (Level 4). Some vials are designed with a special stopper that allows for more precise reconstitution and less loss of product. In addition, some syringes have been designed to ensure a more accurate injection of the product, reducing the risk of over‐injection and therefore reducing wastage.

Reducing wastage during botulinum toxin extraction and administration is critical in providing cost‐effective and safe treatment for patients. Therefore, it is crucial to educate healthcare providers on the most effective methods of botulinum toxin extraction and administration, ensuring patients receive optimal care while minimizing wastage. These techniques can reduce the amount of product lost during the reconstitution process, leading to cost savings for patients and practitioners, and to a more sustainable use of resources. Further research is needed to verify the effectiveness of these techniques and to identify new ways to minimize botulinum toxin wastage.

## CONFLICT OF INTEREST STATEMENT

We acknowledge that we have considered the conflict of interest statement included in the “Author Guidelines.” We hereby certify that, to the best of our knowledge, no aspect of our current personal or professional situation might reasonably be expected to significantly affect our views on the subject we are presenting.

## Data Availability

The data that support the findings of this study are available from the corresponding author upon reasonable request.
